# A Proposed COVID-19 Testing Algorithm

**DOI:** 10.1017/dmp.2020.218

**Published:** 2020-06-24

**Authors:** Alexander Hart, Michelangelo Bortolin, Oluwafunbi Awoniyi, Fahad Alhajjaj, Gregory R. Ciottone

**Affiliations:** Beth Israel Deaconess Medical Center, Emergency Medicine, Boston, MA; Servizio Emergenza Territoriale 118, Torino, Italy; Unaizah College of Medicine at Qassim University, Emergency Medicine, Unaizah, Saudi Arabia

**Keywords:** communicable diseases, epidemiological monitoring, pandemics, public health, policy making

## Abstract

The 2019 coronavirus disease (COVID-19) pandemic has led to physical distancing measures in numerous countries in an attempt to control the spread. However, these measures are not without cost to the health and economies of the nations in which they are enacted. Nations are now looking for methods to remove physical distancing measures and return to full functioning. To prevent a massive second wave of infections, this must be done with a data-driven methodology. The purpose of this article is to propose an algorithm for COVID-19 testing that would allow for physical distancing to be scaled back in a stepwise manner, which limits ensuing infections and protects the capacity of the health care system.

As the current coronavirus disease (COVID-19) pandemic has progressed, it has forced the majority of the world’s nations to combat the spread via physical distancing of their populations. However, these non-pharmacological interventions take a toll on the populations and economies of the nations where they go into effect.

## THE MEDICAL AND MENTAL HEALTH TOLL

Physical distancing has already been linked with decreased admissions for myocardial infarction (MI) and worsening outcomes in ST-elevated MI, and is likely linked with poor outcomes in many other chronic diseases.^1,2^ Given the link between unemployment and mental health disorders, we must also be concerned with the potential rise of suicides, addiction, and domestic abuse. Essential workers [EW] who work in facilities that treat those with COVID-19 are at particularly high risk for mental health disorders related to the traumatic events they witness.^3^ Physical distancing prevents EW and the general population from engaging in social behaviors, which for many is a method of defusing the tension inherent in traumatic events, such as living through a pandemic.

## THE ECONOMIC TOLL

As physical distancing measures have been in place, many sectors of the economy have closed or slowed significantly. The US unemployment rate jumped from 4.4% to 14.7% in April 2020, having been below 4% for almost a year prior to that. This is in addition to a doubling over the course of 1 month of the population who work part-time due to reductions in their hours or lack of full-time job availability.^4^ The International Monetary fund projects that world gross domestic product will drop 3% in 2020, after growing 2.9% in 2019, with advanced economies such as the United States, Japan, Germany, and Italy dropping an average of 6.1%.^5^ These drops in employment and productivity have far ranging consequences and can cause pressure to be applied to public health officials to roll back physical distancing measures.

## EFFECTIVENESS

The effectiveness of physical distancing policies has been proven during the 1918 Spanish flu epidemic.^6^ Recent research and modeling are beginning to demonstrate the effectiveness of physical distancing measures in the current pandemic, with current estimates of roughly 3.1 million lives saved across several European countries and 4.8 million fewer cases occurring in the United States.^7,8^ Despite this, less research has been performed showing how nations can most safely roll back these measures as the pandemic becomes better controlled.^9^

## CURRENT GUIDELINES

Recently, the US Government published a “Framework for Reopening America,” which contains criteria guidelines for states to reopen economically.^10^ The criteria for this process of reopening the economy include, “confidence that incidence of infection is genuinely low” and “a surveillance system that is well functioning and capable of promptly detecting any increase in incidence.” These criteria aim to provide a balance between physical distancing to protect the health system and an open society that provides for as much economic freedom as possible. The framework recommends which groups to prioritize for COVID-19 testing; however, this guidance leaves much to be desired in its lack of specificity.

## MODELING

Current data coming from countries, such as South Korea and Germany, suggest that a large portion (up to 60%, in some estimations) of those infected with COVID-19 will be asymptomatic carriers.^11^ When modeling the pandemic, this suggests the need to use the Susceptible-Exposed-Infectious-Removed (S-E-I-R) model of disease, which categorizes the population into 4 groups based on infectivity. The *Susceptible* group has not been infected and are still able to become infected if exposed to COVID-19. The *Exposed* group includes those who have become infected and are infectious but have not yet begun to have symptoms. The *Infectious* group includes those who are asymptomatic carriers and those who have current symptoms, and thus are able to infect others. The *Removed* group includes those who have recovered and may become immune and those who are deceased and therefore no longer infectious.^12^

Identifying these susceptible and recovered groups is vital to the downregulation of physical distancing measures, as it will allow for public health officials to predict future numbers of infections and scale back physical distancing accordingly. While the need to scale up nasopharyngeal swab testing (and identify the infectious group) of the asymptomatic population has been discussed extensively, less attention has been paid to the need to define the recovered population.

Serology testing has been touted previously as a method to determine this population due to the ability to detect antibodies in the bloodstream, thus identifying recovered populations who never manifested symptoms, in addition to those who had a positive nasopharyngeal swab.^13^ As of April 23, there were 4 serologic tests with Federal Drug Administration (FDA) Emergency Use Authorizations in the United States, though there are significantly more tests being brought to market.^14^

After the recovered population is identified, 1 method to decrease infections would include identifying a cohort of those individuals who have recovered from COVID-19 in jobs with a higher chance of future exposure. This is based on the presumption that, like most infections, when recovered from COVID-19, a patient has immunity via their antibody production. While this is still an open question for COVID-19, early animal studies have suggested this to be true.^15^ For this plan to succeed, there must be a clearly defined process to provide focused testing on a massive scale, so that the recovered population can be fully identified.

## METHODS

Here, we propose a testing algorithm to identify the susceptible, infectious, and recovered groups, so that physical distancing measures can be relaxed in a graded, data-driven manner. The algorithm could be applied only to EW in the beginning, and as soon as testing capability is increased, applied more broadly to the general population. The goals of this testing algorithm are fourfold:

1. Identify asymptomatic, exposed EWs to prevent them from spreading COVID-19.
2. Identify recovered EWs and move this cohort in areas where COVID-19 exposure is highest.
3. When capacity allows for more widespread testing, identify the recovered lay population who can return to work first, and begin the work of restarting the economy.
4. Ensure the safety of EWs who are still susceptible, which can additionally improve morale among the EW population.

*Essential workers* are defined as those in job functions that are vital to the functioning of basic public services. They are those who have continued to work in-person at their facility, despite the risk to their personal health and that of their family. While different nations and regions have included other functions that they found essential, all include those who work in health care; food production and supply chain; and public infrastructure, such as water, sewer, and power.

### Identify the Asymptomatic Exposed Population

Up to 44% of spread of COVID-19 is likely related to spread from asymptomatic individuals.^16^ Thus, a major goal of this algorithm is to identify the exposed. This task requires frequent testing of the population even without symptoms or known exposure. Routine testing is more powerful the more frequently it occurs. However, daily testing is likely too resource-intensive and onerous on the population, to be feasible.

Ideal scenarios would additionally feature frequent testing of all non-recovered individuals. However, given the current limits to testing in the United States and many other countries, this is also unlikely to be feasible in a reasonable time period. Thus, a strategy of nasopharyngeal testing was chosen, which focuses on those with the highest likelihood of having COVID-19, those with a known exposure or symptoms. This initial testing pattern could then be upgraded to routine testing every 1–2 weeks as test capacity increased.

### Identify the Recovered Population of Essential Workers

If a cohort of recovered EWs can be identified, they can work shifts in areas of highest risk exposure such as the emergency department, intensive care unit, or in the non-health-care setting, areas of the workplace that are not amenable to physical distancing. This would decrease the exposure for the susceptible population, who could then be shifted to lower risk settings.

To identify recovered EWs, serology testing will be used to identify those with evidence of prior infection and immunity. Those who initially test positive for immunity would be certified as immune so as to allow for full work and economic activities, and potentially be moved in a cohort to high-exposure work areas. However, as EWs continue to work, there will be likely further asymptomatic infections leading to immunity. To identify this population, repeat serologic testing is necessary. Testing every 2 weeks was chosen to match the reported incubation period of COVID-19. This way, a newly immune EW could be identified as soon as possible.

### Identify the Recovered Lay Population

As physical distancing measures are relaxed, more and more of the populace will be spending time in public where they have increased exposure. This algorithm can be applied to any number of the populace, providing that a testing infrastructure is present for both nasopharyngeal swab and serologic testing.

As the lay population is identified as immune, they can also be certified for full work and economic activity. This would lead to a gradual increase in the population able to work as more and more people test positive for immunity. Multiple methods have been proposed for this certification, including “Immunity Cards/Passports.” The method of certification is beyond the scope of this algorithm, but it would need to be integrated into the administration of the public health interventions of the region using the algorithm. Concerns about discrimination of those without “Immunity Cards/Passports” and risks to those who had a false-positive test becoming less careful about disease avoidance make these types of interventions problematic.

### Ensure the Safety of the Susceptible Population

The susceptible population remains at high risk of COVID-19 infection for as long as they have exposure to infected or exposed persons. EWs have continued to be exposed throughout the pandemic. As an immune population of EWs is identified, those who are immune can take on the highest exposure jobs. This would decrease the total exposure for those who are still susceptible.

This would blunt the risk of large boluses of new cases as physical distancing measures are scaled back. As a greater population moves about in public, the total exposure to COVID-19 will increase simply due to the volume of persons unidentified as exposed/infected with whom individuals will interact. By identifying the recovered individuals and moving this cohort in locations of high exposure (eg, customer interaction, emergency departments, intensive care), those who are still susceptible can be allowed to function without increasing their exposure.

## RESULTS

Given the current levels of testing available, this algorithm focuses first on EWs. All persons who are able to continue physical distancing would continue to do so, which serves several purposes. First, this would limit their exposure, protecting the health care system from sudden overload. Second, it would save tests for use on EWs until testing capability is increased. Finally, it allows for the development of herd immunity.

The algorithm then begins by providing serology testing every 2 weeks on the EWs who return to work. This time frame was chosen as it is the approximate incubation period of the COVID-19. Those who are shown to have previous infection and immunity will then no longer need further testing. Those who are not immune will continue to work with appropriate personal protective equipment and be re-tested every 2 weeks.

If an EW has a concern with COVID-19 exposure or develops symptoms, the EW would get nasopharyngeal testing for active COVID-19 infection. Those with negative testing will re-enter into the normal serologic testing pathway. EWs who are confirmed as positive for COVID-19 infection via nasopharyngeal swab will be isolated for 14–21 days until their infection has passed. At the end of this time, the EWs will again undergo serologic testing. If they are then proven to be immune, they will be certified for full activity and will exit the algorithm. If they do not show signs of immunity on serologic testing, they will re-enter the previous series of testing every 2 weeks. When testing is available, the same algorithm could be used for the general public.

## DISCUSSION

This testing algorithm relies heavily on the idea that serology testing for IgM and IgG correlates with immunity to recurrent infection. However, as this question remains unanswered, further research will be required to validate the use of this algorithm. Additionally, as with all testing, the lower the prevalence of COVID-19 antibodies, the worse the positive predictive value (PPV) of the test becomes. The US Centers for Disease Control and Prevention (CDC) currently estimate the PPV for a serology test in a population with 5% prevalence to be 48.6%, with 2 positive tests having a PPV of 94.5%, which may mean that lower prevalence areas may require 2 positive serology tests to clear an EW for full work.^17^

Of note, there is little evidence to date to prove the negative predictive value [NPV] of a nasopharyngeal swab. Given the variety of different tests being used worldwide, it would be difficult to make universal recommendations based on the NPV, even if it was known for a single test. Some localities have required serial negative swab tests (2 or 3) before declaring a patient as negative for COVID-19. While this algorithm does not address these questions of NPV, local public health departments will need to decide on the need for serial testing based on estimations of their own test characteristics (including both sensitivity and specificity) and risk to the population of a false negative or false positive. As the prevalence of COVID-19 goes down, the PPV of nasopharyngeal swabs will also drop, leading to concerns about increasing percentages of positive tests leading to incorrect isolation.

Persons who are able to work from home with minimal interruptions should continue to until societal levels of recovered persons demonstrate the development of herd immunity. The concept of herd immunity is that if a sufficiently high percentage of the population is immune to an infectious disease, this will provide fewer opportunities for it to spread, giving additional protection against transmission to those who are still susceptible. The incidence of infection will decline if the immune proportion is > ([R_0_ -1]/R_0_).^18^ Thus, in the case of COVID-19, for which the CDC quotes an R_0_ of 2.2-2.7,^19^ the immune proportion must be between 55.5% and 63.0%. When this proportion is reached, either via serologic testing or vaccination, the community in question could further relax physical distancing measures.

This algorithm will face multiple issues of practicality, which may make it more viable in some localities than in others. Testing the full population will require vast quantities of virologic and serologic tests. Given that many places have struggled to keep reagent and swabs in stock, some may be able to extend this algorithm to only a small portion of EWs, such as medical providers and first responders. Hopefully, as the production of all the components of testing increases, more and more localities will be capable of expanding the algorithm to include their overall population. The algorithm would also require personnel to perform and run the tests. Given the development of many drive-by testing sites, perhaps these could be expanded to perform asymptomatic nasopharyngeal testing and serologic testing for those who qualify in the algorithm.

Mass vaccination is the most likely method by which the population will reach herd immunity. While the hope is that a viable vaccine will be mass produced and available to the general public in the near future, this algorithm can be used to accelerate the release of physical distancing measures until this occurs.

Finally, as the population is identified by its immune status, care must be taken to avoid public backlash. Public health officials would be wise to understand the risk that some who are still susceptible could intentionally expose themselves to COVID-19 in an attempt to become immune and be allowed to return to work (similar to the “chickenpox parties” held in the United States prior to the varicella vaccination). Interventions to educate the public to avoid this or to enforce penalties for those who intentionally expose themselves are among the potential methods to address this problem.

## CONCLUSIONS

We are currently confronted with a public health dilemma that has not been experienced since the early 20th century: How does a society safely progress from a state of physical distancing to that of normal operations in the presence of a pandemic infectious disease? While there are few historical examples to draw from, a well thought out algorithm of testing to identify key populations can aid in the stepwise loosening of restrictions. This algorithm is based on the assumption that serologic testing can identify those now immune to re-infection with COVID-19. We propose large-scale government financing of research to investigate this question of conveyance of immunity. If verified, this testing regimen could then be applied first to EWs and then to the general population when testing capabilities become available.
