# A Proposed COVID-19 Testing Algorithm–Corrigendum

**DOI:** 10.1017/dmp.2020.306

**Published:** 2020-10

**Authors:** Alexander Hart, Michelangelo Bortolin, Oluwafunbi Awoniyi, Fahad Alhajjaj, Gregory R. Ciottone

**Keywords:** communicable diseases, epidemiological monitoring, pandemics, public health, policy making, corrigendum

The original publication of this article^1^ omitted a graphic representation of the proposed testing algorithm. Here is that figure.

**FIGURE 1:**
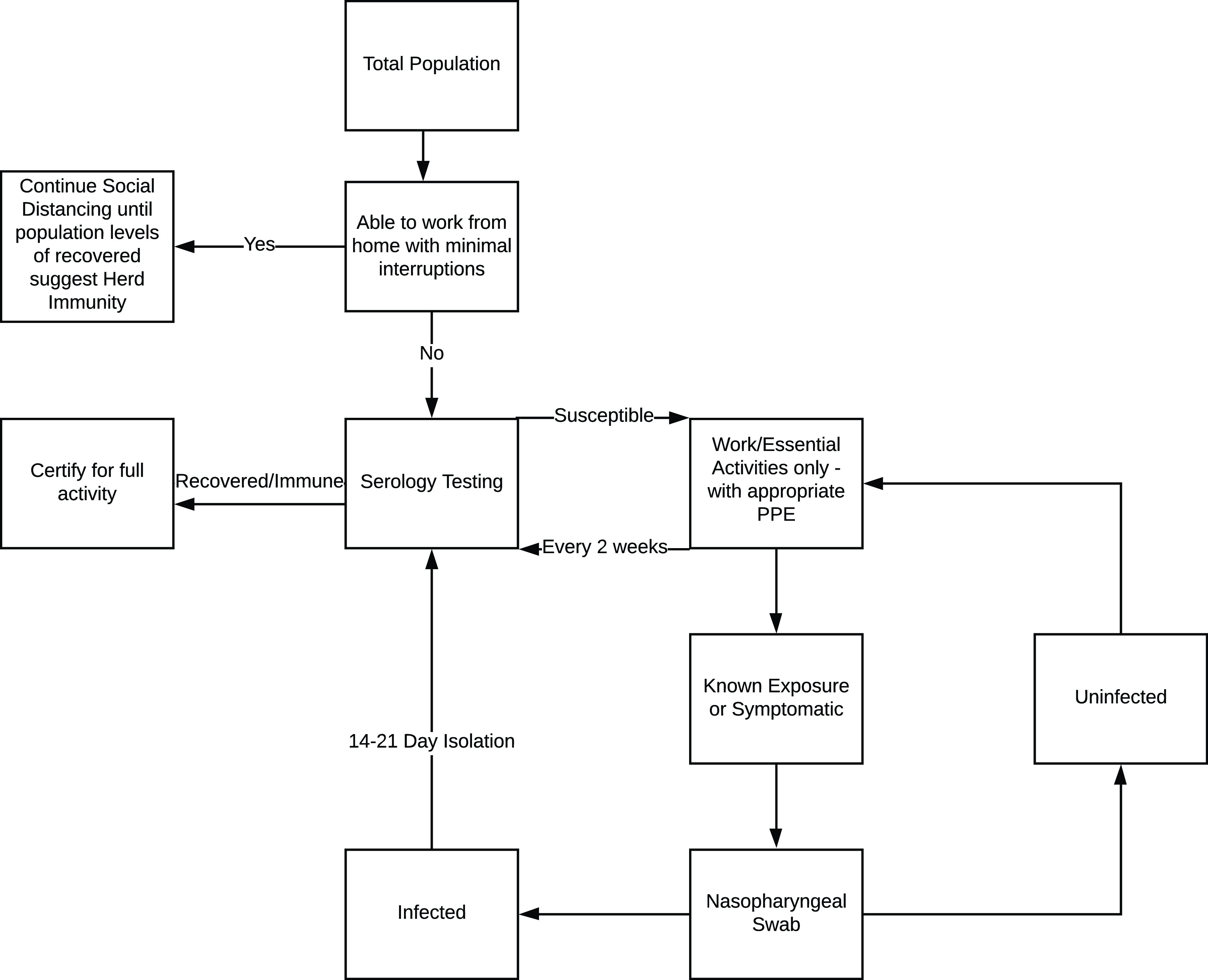
COVID-19 Proposed Testing Algorithm.
